# Comprehensive analysis of publications concerning combinations of immunotherapy and targeted therapies for hepatocellular carcinoma: a bibliometric study

**DOI:** 10.3389/fimmu.2025.1476146

**Published:** 2025-02-12

**Authors:** Biling Gan, Lei Wu, Shunan Zhou, Zhihong Chen, Fan Wu, Lianqun Xu, Zhenrong Chen, Honghui Ma, Peijia He, Dan Fang, Ning Shi

**Affiliations:** ^1^ Department of Hepatobiliary Surgery, Guangdong Provincial People’s Hospital (Guangdong Academy of Medical Sciences), Southern Medical University, Guangzhou, China; ^2^ Guangdong Cardiovascular Institute, Guangdong Provincial People’s Hospital, Guangdong Academy of Medical Sciences, Guangzhou, China; ^3^ Department of Liver Surgery, Peking Union Medical College Hospital, Chinese Academy of Medical Science and Peking Union Medical College, Beijing, China; ^4^ School of Medicine South China University of Technology, Guangzhou, Guangdong, China

**Keywords:** hepatocellular carcinoma, immunotherapy, targeted therapy, combination therapy, bibliometric study, VOSviewer, CiteSpace

## Abstract

**Background:**

Hepatocellular carcinoma (HCC), a prevalent malignancy, is often diagnosed at advanced stages. Recent advances have integrated immunotherapy with targeted therapy, significantly improving treatment outcomes. This study provides a bibliometric overview of these therapeutic combinations, evaluating their development and impact.

**Methods:**

A rigorous selection process was applied to relevant literature from Web of Science, followed by in-depth bibliometric analyses— including timeline visualization, burst detection, and co-occurrence analysis—using CiteSpace and VOSviewer. This approach offered insights into the contributions of countries, institutions, authors, journals, references, and key terms within the field.

**Results:**

A total of 506 studies published between 2014 and 2023 were included, with all articles in English. Mainland China dominated the publication output, contributing 40% (N = 202), followed by significant contributions from the United States and Japan. Kindai University led institutional contributions, accounting for 7.9% of the total (N = 40). The authors Kudo Masatoshi and Hatanaka Takeshi were the most prolific, each with nine publications. The journal Cancers emerged as the top publisher, with 48 relevant articles and an Impact Factor of 5.2 in 2022. A co-citation network analysis traced the evolution of immunotherapy and targeted therapy combinations in HCC treatment. Early research primarily focused on angiogenesis, dendritic cells, and expression markers, while recent trends have shifted towards phase III trials, adverse reactions, and checkpoint inhibitors, underscoring the field’s dynamic progression.

**Conclusion:**

Future research will expand on the pathological mechanisms underlying these therapies and novel interventions and combination strategies. Addressing adverse events and treatment discontinuation will remain central to advancing clinical applications.

## 1 Introduction

Primary liver cancer is the sixth most prevalent cancer worldwide and the fourth leading cause of cancer-related mortality. Hepatocellular carcinoma (HCC) accounts for approximately 75% of all primary liver cancer cases (1). Upon diagnosis, only 30-40% of patients with HCC are candidates for radical surgery, while the majority receive systemic treatment due to the asymptomatic nature of the disease and the lack of specific biomarkers in its early stages (2). Systemic therapies, including chemotherapy, targeted therapy, and immunotherapy, play a pivotal role in managing intermediate and advanced HCC (3). Despite significant progress in chemotherapy and targeted therapies over recent decades, HCC continues to be highly susceptible to drug resistance, recurrence, metastasis, and poor prognosis during treatment (4). With ongoing advancements in immune checkpoint inhibitors (ICIs), the combination of molecular targeted agents and ICIs has shown promising efficacy in HCC treatment, becoming a major area of focus (5, 6).

Recently, an increasing number of studies have been published on the combination of targeted therapy and immunotherapy for HCC. However, no comprehensive literature analysis on this topic has been conducted thus far. Bibliometric analysis, which involves the quantitative examination of knowledge carriers (typically literature) within a particular field using mathematical and statistical methods, helps assess research trends, elucidate the knowledge structure, and forecast potential breakthroughs. Current bibliometric studies predominantly employ tools such as CiteSpace (7) and VOSviewer (8). This approach has been widely applied across various fields (9–12), but no bibliometric study has specifically mapped the knowledge related to the combination of targeted therapy and immunotherapy for liver cancer (13–15). Therefore, the primary objective of this study is to provide an in-depth evaluation of the current state of targeted and immunotherapy combinations for liver cancer and offer researchers clear guidance for future research directions.

## 2 Materials and methods

### 2.1 Data sources and search strategy

The Web of Science (WoS) Core Collection databases were accessed to retrieve relevant English-language articles on the combination of immunotherapy and targeted therapy for HCC published up until December 31, 2023. The search keywords included “primary liver cancer,” “targeted therapy,” and “immunotherapy,” with additional keywords provided in **Supplementary File S1**. Titles and abstracts of the retrieved articles were manually assessed according to the following inclusion criteria: (1) relevance to immunotherapy and targeted therapy combinations in HCC treatment, (2) publication type (research papers, clinical trials, meta-analyses, or reviews), and (3) English language. Exclusion criteria included: (1) treatment combinations involving chemotherapy, radiotherapy, interventional therapy, radiofrequency ablation, or surgery, (2) single modality targeted therapy or immunotherapy, and (3) inaccessible full texts or abstracts. Relevant information (title, year of publication, authors, country, attribution, journal, keywords, and abstract) from the included literature was downloaded in.txt format.

### 2.2 Bibliometric analysis

Data analysis was performed using CiteSpace 6.3.R1, with a time range from January 1, 2014, to December 31, 2023, divided into 10 annual time slices. The analysis employed Pathfinder, pruning sliced networks, and merging network pruning methods. The k-value of the g-index was adjusted based on the data values, while other parameters remained at their default settings. Nodes such as authors, institutions, countries, and keywords were selected for visual analysis. VOSviewer 1.6.20 was utilized to analyze country, institution, author contributions, and research hotspots using default parameters. Data processing was carried out in Microsoft Excel, followed by the establishment of index models.

## 3 Results

### 3.1 Literature collection and publication prediction

**Figure 1:**
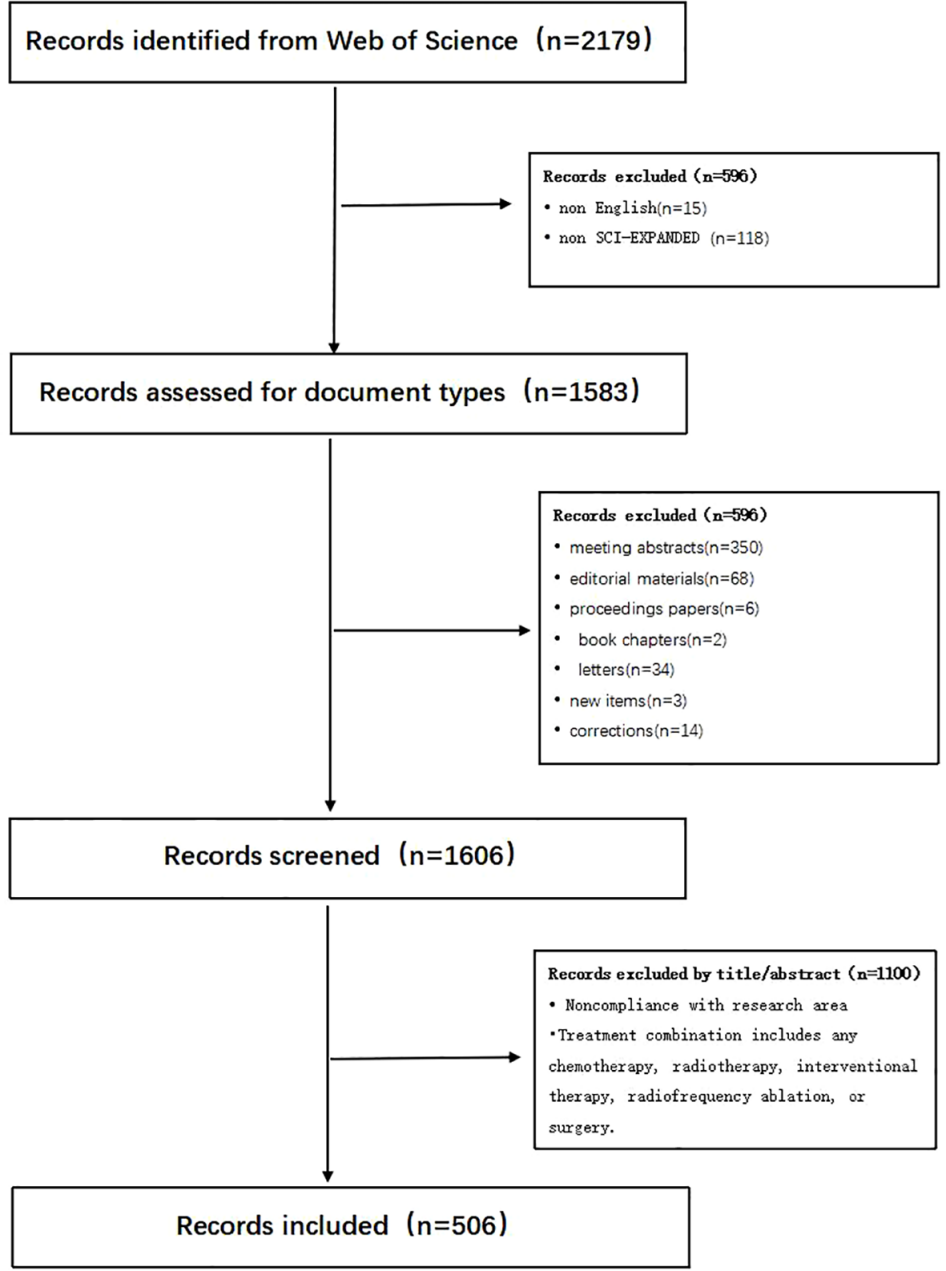
Literature search process.

**Figure 2:**
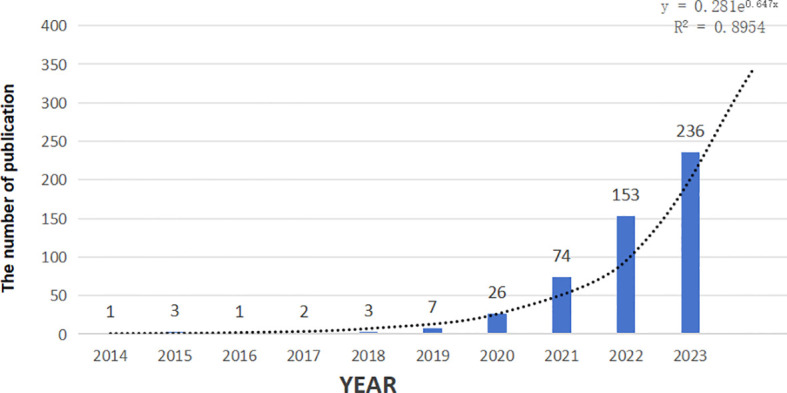
Polynomial curve fitting of publication growth in immunotherapy and targeted therapy combinations for HCC.

A total of 2,179 articles focusing on the combination of immunotherapy and targeted therapy in HCC treatment were retrieved from the WoS Core Collection using the described search strategies. After applying stringent inclusion and exclusion criteria, 506 articles were included in the final analysis, as shown in (**Figure 1**). Publications on this topic have exhibited a significant upward trend from 2014 to 2024, with an estimated 346 articles expected in 2024, based on the fitting curve (**Figure 2**).

### 3.2 Analysis of national publications

**Figure 3:**
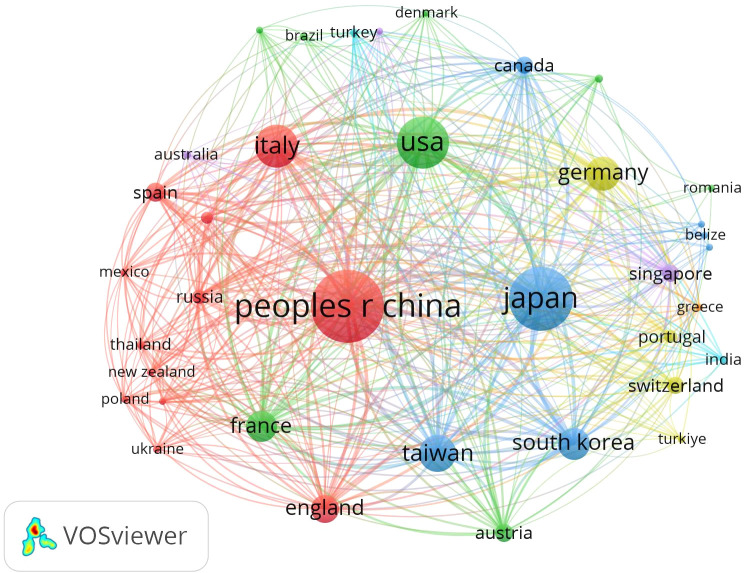
Network map showing countries/regions involved in research on immunotherapy and targeted therapy combinations for HCC.

The 506 included articles were published by authors from 126 countries and regions (**Figure 3**). Mainland China led the contributions, publishing 35.4% of the articles (179 publications, centrality = 0.05). Taiwan contributed 23 articles (4%) with a centrality of 0.15. Other leading contributors included Japan (119 articles, 23.5%) and the United States (63 articles, 12.5%). Mainland China also had the highest citation count (N = 204), followed by Japan (N = 153) and the United States (N = 102). France had the highest centrality score of 0.62 (**Table 1**).

**Table 1:** Top 15 countries/regions involved in research on immunotherapy and targeted therapy combinations in relation to HCC.

Countries	Counts	Percent	Centrality	Citation Counts
China	179	35.40%	0.05	204
Japan	119	23.50%	0.05	153
USA	63	12.50%	0.34	102
Italy	40	7.90%	0.26	72
France	25	4.90%	0.62	37
Tai Wan	23	4.00%	0.15	51
South Korea	15	2.90%	0.13	38
Germany	14	2.70%	0.00	45
England	10	1.90%	0.39	30
Singapore	4	0.80%	0.21	12
Canada	4	0.80%	0.09	11
Spain	3	0.60%	0.30	14
Australia	3	0.60%	0.11	13
Belgium	3	0.60%	0.11	6
Switzerland	1	0.20%	0.00	11

### 3.3 Analysis of institution publications

**Figure 4:**
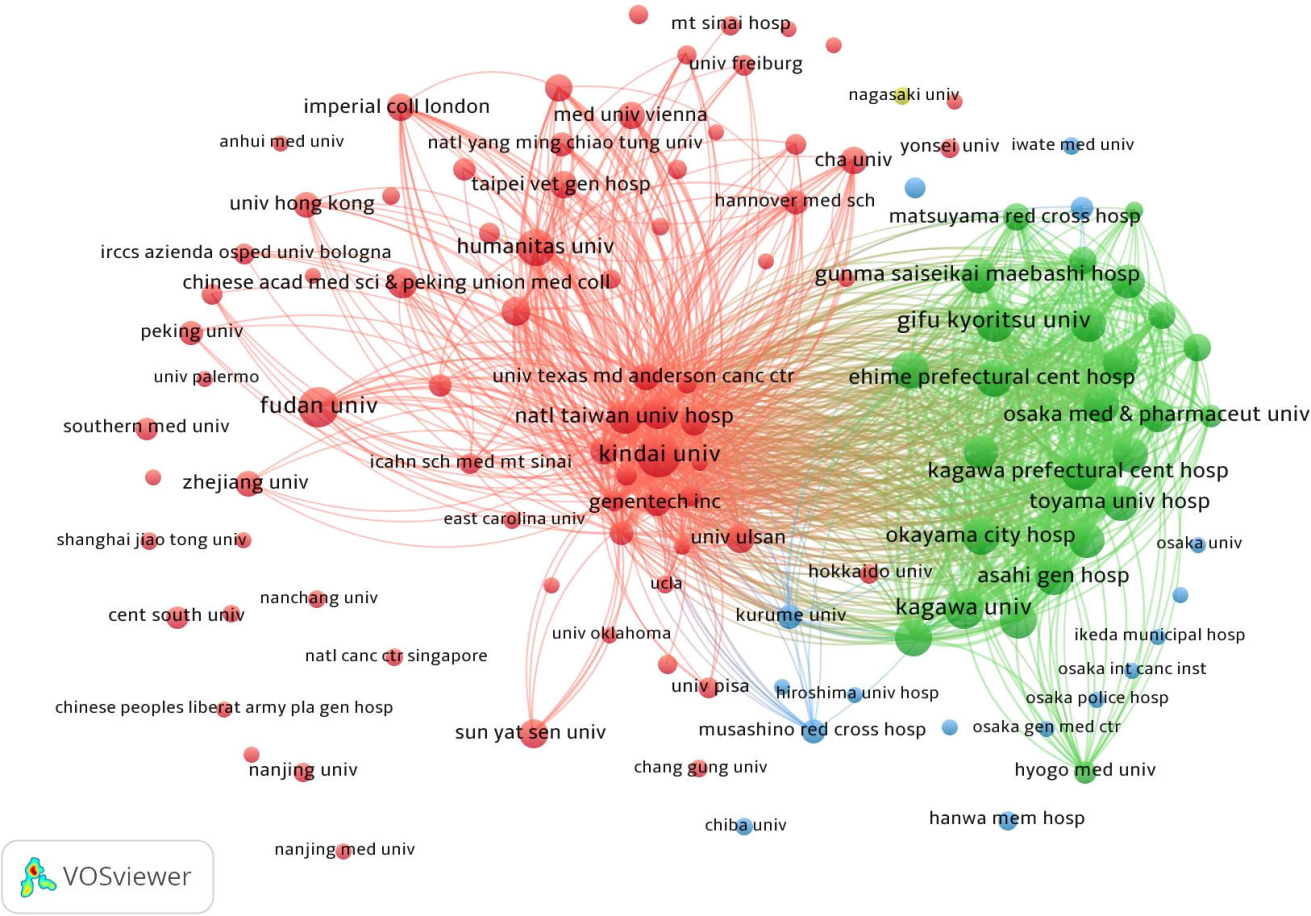
Network map showing institutions involved in research on immunotherapy and targeted therapy combinations for HCC.

The analysis included data from a total of 2,679 institutions (**Figure 4**). Kindai University published the most articles (N = 40), while Matsuyama Red Cross Hospital and Kagawa University each contributed 36 articles (**Table 2**). The top three institutions with the most publications are all based in Japan. Harvard Medical School exhibited the highest centrality score of 0.58 (**Table 2**). Institutions with higher centrality were predominantly located in the United States and mainland China.

**Table 2:** Top 10 institutions involved in research on immunotherapy and targeted therapy combinations in relation to HCC.

Institutions	Citation Counts	Institutions	Centrality
Kindai University (Kinki University)	40	Harvard Medical School	0.58
Matsuyama Red Cross Hospital	36	UTMD Anderson Cancer Center	0.39
Kagawa University	36	National Taiwan University	0.32
Nippon Medical School	35	National Taiwan University Hospital	0.27
Ogaki Municipal Hospital	34	Sun Yat Sen University	0.26
University of Toyama	34	Seoul National University (SNU)	0.25
Hyogo College of Medicine	33	UNICANCER	0.21
Teine Keijinkai Hospital	33	Universite de Rennes	0.17
Ehime University	33	Gustave Roussy	0.16
Hamamatsu University School of Medicine	33	Azienda Ospedaliero Universitaria Pisana	0.14

### 3.4 Author analysis

A total of 3,669 authors contributed to the research in the included articles. Kudo Masatoshi and Hatanaka Takeshi were the most prolific authors, each publishing nine papers, while Tada Toshifumi contributed seven (**Table 3**). Co-citation analysis revealed that Kudo Masatoshi (N = 44), Tada Toshifumi (N = 38), and Nakamura Shinichiro (N = 38) were the top three authors in terms of co-citations.

**Table 3:** Top 10 authors and co-cited authors involved in research on immunotherapy and targeted therapy combinations in relation to HCC.

Co-cited Author	Counts	Author	Counts
Kudo, Masatoshi	44	Kudo, Masatoshi	9
Tada, Toshifumi	38	Hatanaka, Takeshi	9
Nakamura, Shinichiro	38	Tada, Toshifumi	7
Ogawa, Chikara	37	Hiraoka, Atsushi	6
Tani, Joji	36	Fulgenzi, Claudia Angela Maria	4
Ochi, Hironori	36	Persano, Mara	4
Hatanaka, Takeshi	35	Qin, Shukui	4
Atsukawa, Masanori	35	Takada, Hitomi	4
Hiraoka, Atsushi	35	Hsu, Chiun	3
Kumada, Takashi	35	Komatsu, Shohei	3

**Figure 5:**
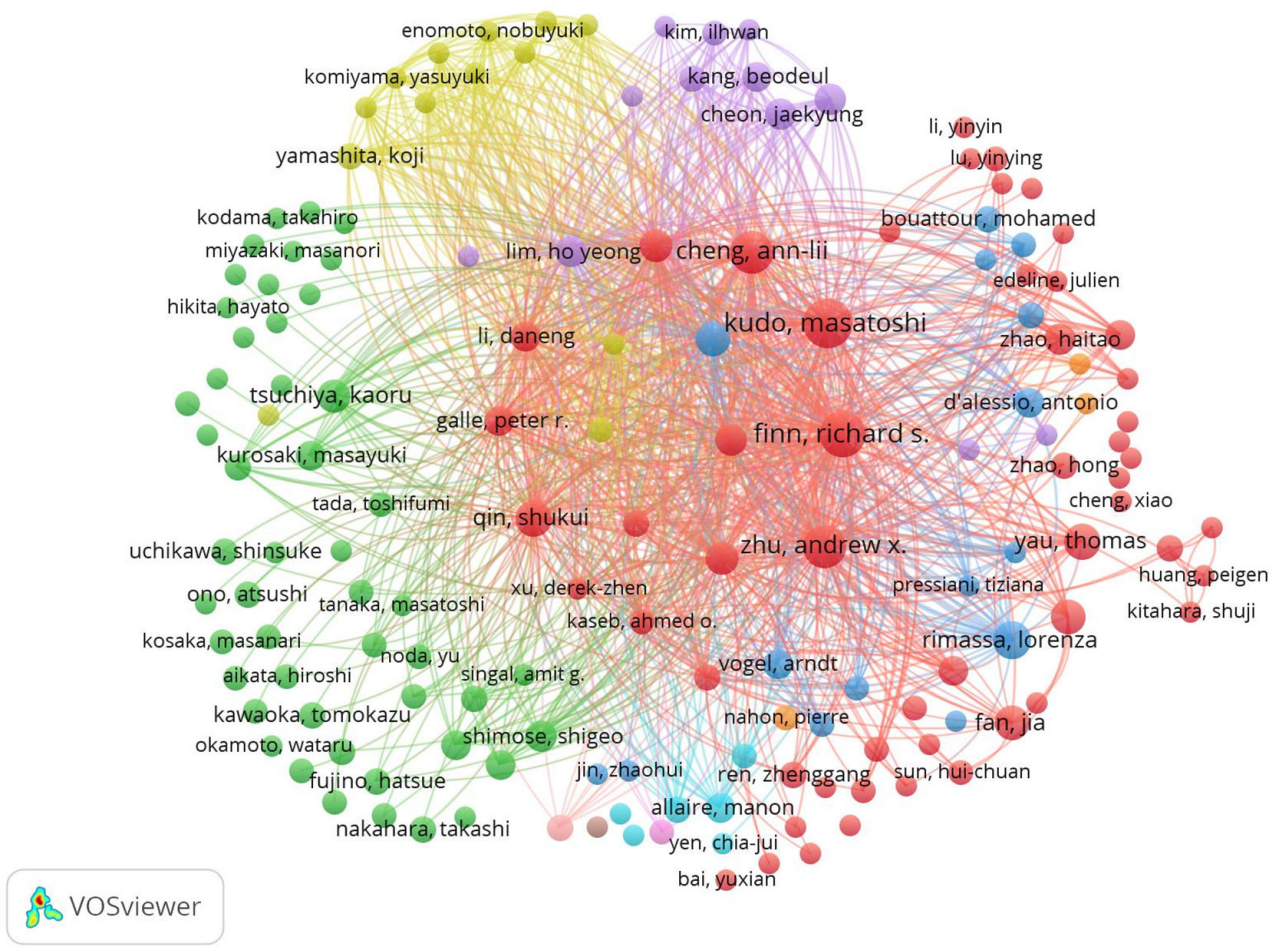
Network map showing authors and co-cited authors involved in research on immunotherapy and targeted therapy combinations for HCC.

VOSviewer was used for network analysis of co-authorship and co-citation among the authors of the selected publications (**Figure 5**). Network nodes represent individual authors, with the size of the circles corresponding to the number of articles published by each author. Co-occurrence relationships are depicted by lines linking the circles. A strong co-occurrence relationship exists between authors and their co-cited counterparts, with authors publishing more frequently generally exhibiting greater co-occurrence relationships with other authors.

### 3.5 Analysis of journals and co-cited academic journals

**Figure 6:**
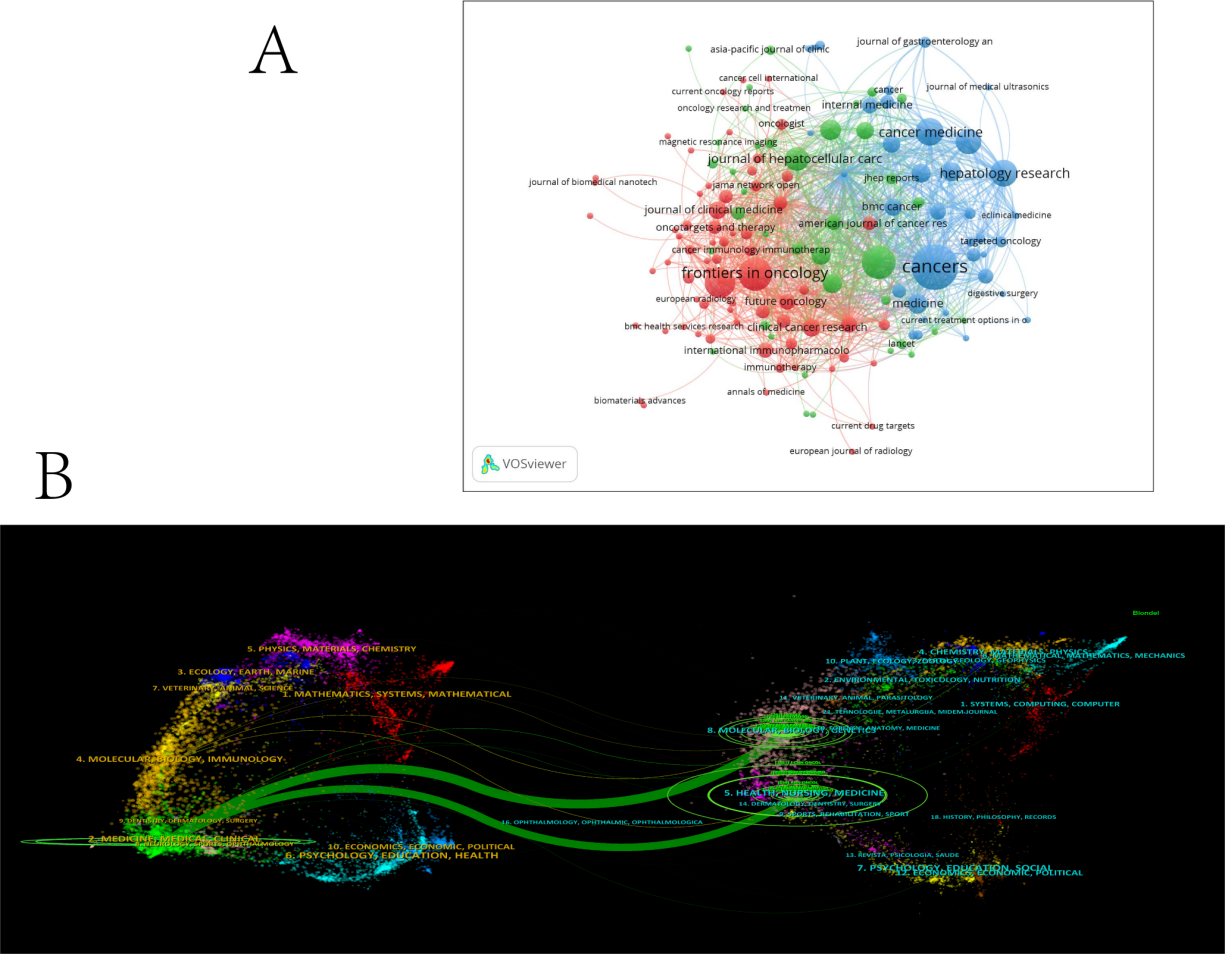
**(A)** Network map showing academic journals involved in research on immunotherapy and targeted therapy combinations for HCC. **(B)** A dual-map overlay of journals related in research on immunotherapy and targeted therapy combinations for HCC.

A total of 147 academic journals published relevant articles on the combination of immunotherapy and targeted therapy in HCC treatment (**Figure 6A**). Cancers led with the most publications (N = 48, IF2022 = 5.2), followed by Frontiers in Oncology (N = 25, IF2022 = 4.7) and Liver Cancer (N = 25, IF2022 = 13.8), both ranking second in terms of publication numbers. Among the top 10 journals contributing the most relevant articles, 50% are based in Switzerland, with 30% from the United States. Liver Cancer ranked highest in Impact Factor (IF2022 = 13.8) among journals with over 20 articles published, followed by Frontiers in Immunology (N = 20, IF2022 = 7.3) (**Table 4**).

**Table 4:** Top 10 academic journals involved in research on immunotherapy and targeted therapy combinations in relation to HCC.

Rank	Journal	Count	IF(2022)#	Country
1	CANCERS	48	5.2	Switzerland
2	FRONTIERS IN ONCOLOGY	25	4.7	Switzerland
3	LIVER CANCER	25	13.8	Switzerland
4	FRONTIERS IN IMMUNOLOGY	19	7.3	Switzerland
5	CANCER MEDICINE	17	4	United states
6	HEPATOLOGY RESEARCH	16	4.2	England
7	ONCOLOGY	15	3.5	Switzerland
8	JOURNAL OF HEPATOCELLULAR CARCINOMA	13	4.1	New Zealand
9	HEPATOLOGY INTERNATIONAL	10	6.6	United states
10	MEDICINE	9	1.6	United states

The dual-map overlay in **Figure 6B** illustrates the distribution of topics within the journals. The citing journals are located on the left, and the cited journals are on the right, with labels representing the disciplines covered by each journal. Colored lines represent citation paths from left to right. The vertical axis indicates the number of papers published, and the horizontal axis denotes the number of authors. Two green pathways highlight frequent citations between journals in the molecular/biology/genetics and health/nursing/medicine domains, pointing to their influence on clinical/medical journals.

### 3.6 Analysis of co-citation

**Table 5** lists the top 10 most-cited papers, with the most citations stemming from Atezolizumab plus Bevacizumab in Unresectable Hepatocellular Carcinoma by Richard S. Finn et al. (N = 391). All top 10 papers have been cited more than 100 times.

**Table 5:** Top 10 co-cited references involved in research on immunotherapy and targeted therapy combinations in relation to HCC.

Rank	Reference	DOI	Count
R-1	Finn RS, 2020, NEW ENGL J MED, V382, P1894	10.1056/NEJMoa1915745	391
R-2	Kudo M, 2018, LANCET, V391, P1163	10.1016/S0140-6736(18)30207-1	312
R-3	Zhu AX, 2018, LANCET ONCOL, V19, P940	10.1016/S1470-2045(18)30351-6	156
R-4	Finn RS, 2020, J CLIN ONCOL, V38, P193	10.1200/JCO.19.01307	155
R-5	Abou-Alfa GK, 2018, NEW ENGL J MED, V379, P54	10.1056/NEJMoa1717002	146
R-6	Zhu AX, 2019, LANCET ONCOL, V20, P282	10.1016/S1470-2045(18)30937-9	133
R-7	Finn RS, 2020, J CLIN ONCOL, V38, P2960	10.1200/JCO.20.00808	125
R-8	El-Khoueiry AB, 2017, LANCET, V389, P2492	10.1016/S0140-6736(17)31046-2	119
R-9	Bruix J, 2017, LANCET, V389, P56	10.1016/S0140-6736(16)32453-9	106
R-10	Cheng AL, 2022, J HEPATOL, V76, P862	10.1016/j.jhep.2021.11.030	102

**Figure 7:**
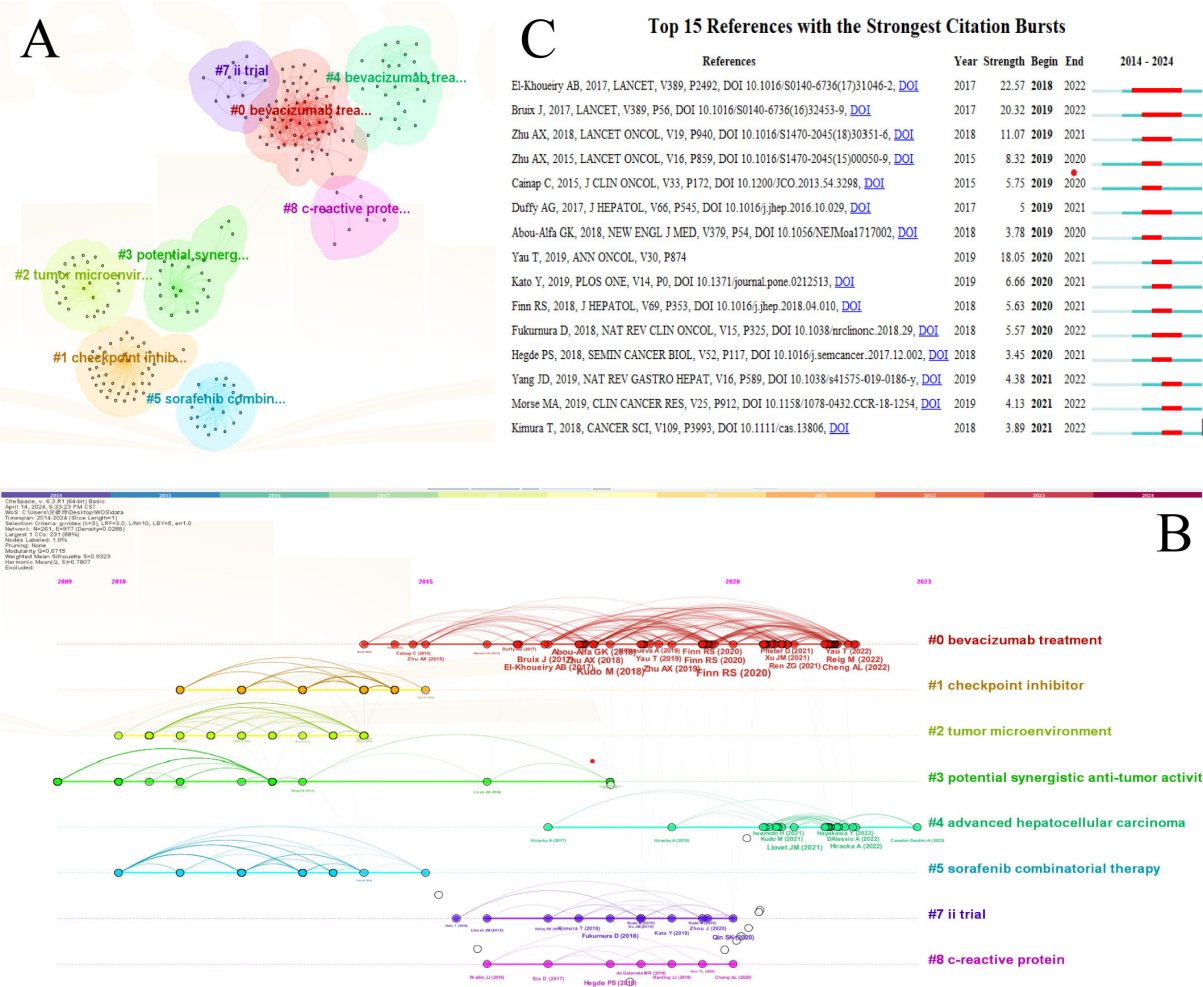
Co-citation analysis of the literature in CiteSpace. **(A)** Cluster analysis of the co-cited literature network. **(B)** Time-axis view of co-citation literature analysis data. **(C)** High-reference outbreak analysis of clustering data.

A co-citation reference network was constructed using CiteSpace, revealing 9 clusters (**Figure 7A**). The clustering analysis identified the most frequently cited keywords, with #0 COMBINING ANTI-VEGF THERAPY as the leading cluster label, followed by #1 COMBINATION THERAPY in second place. A timeline for co-citation references (**Figure 7B**) visually represents the distribution of topics over time. The timeline merges clustering and time-slicing techniques to illustrate research themes’ evolution. Nodes on the timeline, marked by color, represent different years; those on the left are older, and those on the right are more recent. Horizontal lines at the same level indicate the collection of all citations in each cluster, with the labels positioned to the far right. The clusters most adjacent in the timeline include #1 COMBINATION THERAPY, #6 HEPATOCELLULAR CARCINOMA, #7 BEVACIZUMAB TREATMENT, and #8 ADVANCED HEPATOCELLULAR CARCINOMA.

To assess the references with high citation bursts, CiteSpace was used to identify significant bursts in citation frequency. A citation burst indicates a sudden surge in the number of citations, suggesting the research’s growing influence. Among the 15 papers with the strongest citation bursts, El-Khoueiry AB, 2017, LANCET, V389, P2492, DOI 10.1016/S0140-6736 (17)31046-2 (2019-2020, burst = 22.57) and Bruix J, 2017, LANCET, V389, P56, DOI 10.1016/S0140-6736 (16)32453-9 (2019-2020, burst = 20.32) were notable for their high citation numbers (**Figure 7C**).

### 3.7 Analysis of keyword co-occurrence

**Figure 8:**
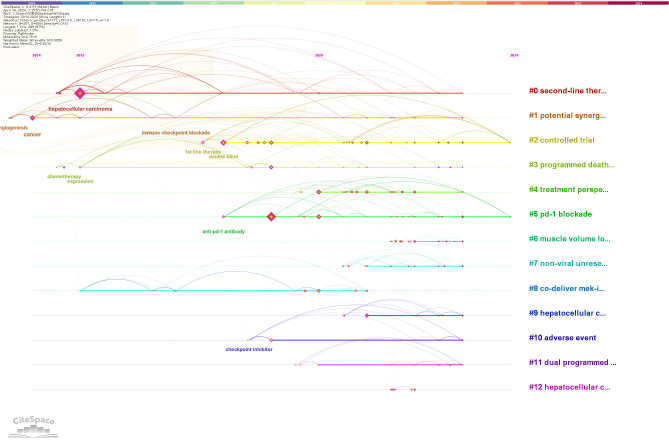
Cluster analysis of keywords in research on immunotherapy and targeted therapy combinations for HCC.

Keyword timeline and clustering analyses serve as effective tools for identifying prominent research themes within a given field. In this study, CiteSpace was employed for clustering keyword data related to immunotherapy in HCC, with cluster numbers based on size, the largest being designated as #0. A total of 13 clusters were identified, which were further analyzed using CiteSpace’s timeline view. These clusters include #0 second-line therapy, #1 potential synergistic anti-tumor activity, #2 controlled trial, #3 programmed death-ligand, #4 treatment perspective, #5 PD-1 blockade, #6 muscle volume loss, #7 non-viral unresectable hepatocellular carcinoma, #8 co-delivery of MEK inhibitors, #9 hepatocellular carcinoma patient, #10 adverse event, #11 dual programmed death receptor-1, and #12 hepatocellular carcinoma. The timeline derived from cluster analysis is shown in **Figure 8**. Four key research areas emerged from co-occurrence analysis: adverse reactions (#6, #10), liver cancer (#7, #9, #12), tumor-targeted immune responses (#1, #3, #5, #8, #11), and the integration of immunotherapy and targeted therapy (#0, #2, #4). Notably, interest in clusters #2, #3, #4, and #5 has grown significantly in recent years and remains high, signaling sustained attention on the combination of targeted therapy and immunotherapy in HCC treatment. As efforts to refine treatment strategies and improve patient outcomes continue, this upward trend is likely to persist.

**Figure 9:**
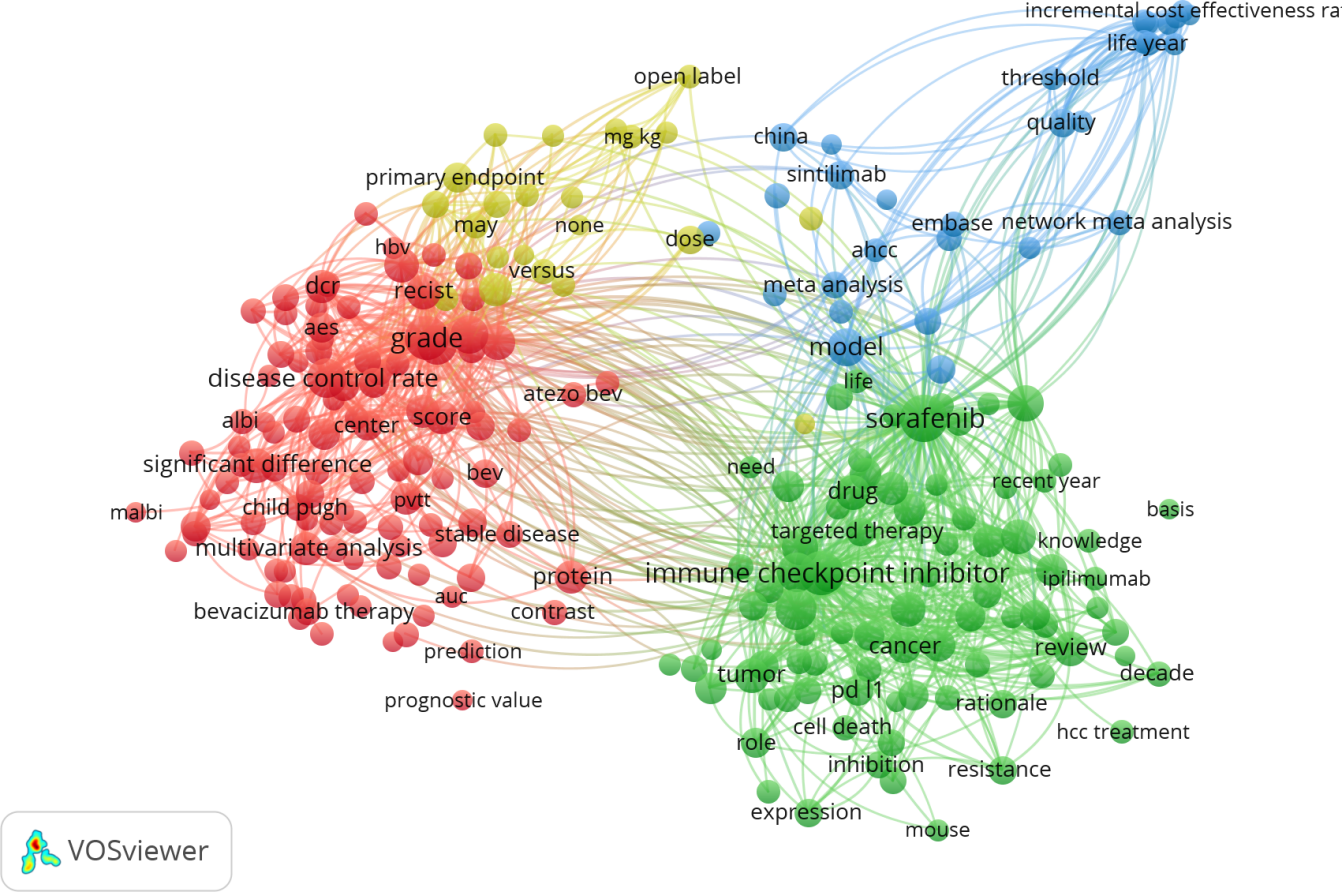
Clustering of keywords over time, presented as a map.

**Figure 10:**
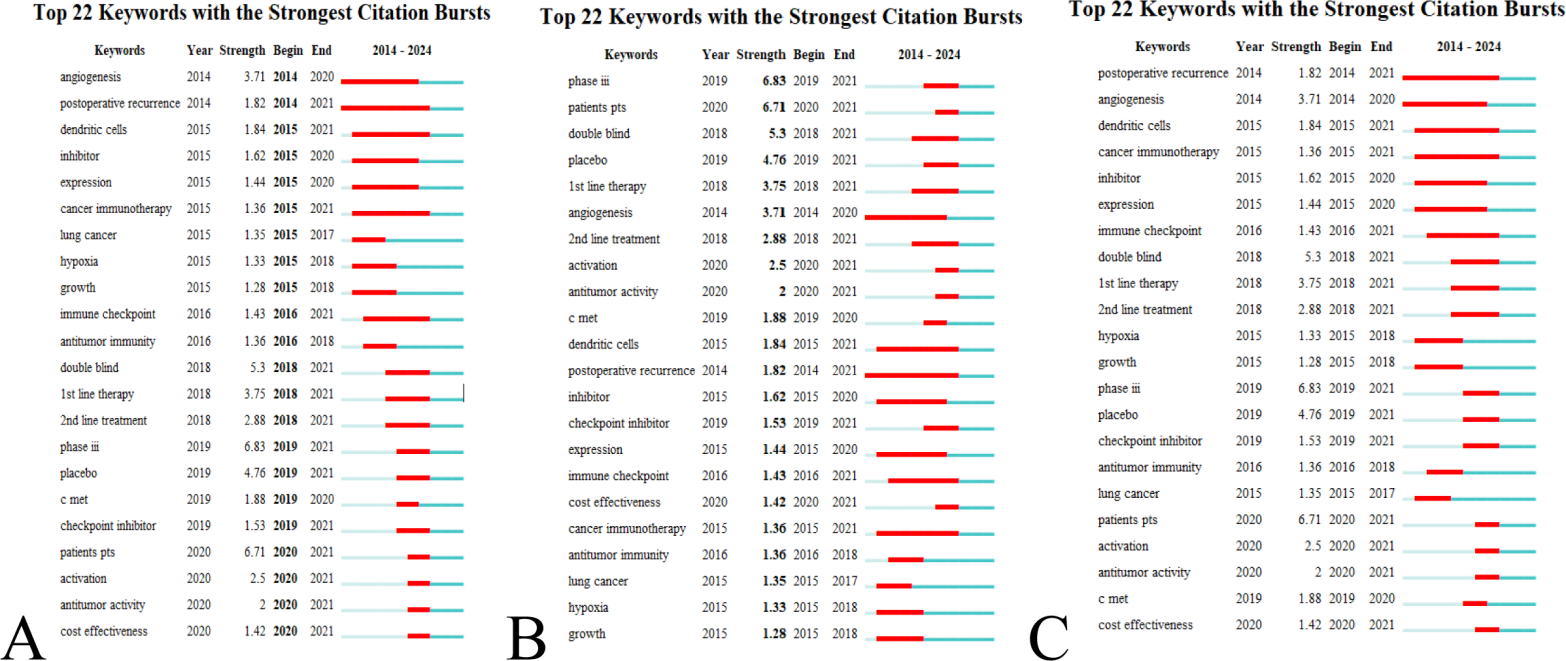
Burst keywords in research on immunotherapy and targeted therapy combinations for HCC. **(A)** Ranking by beginning. **(B)** Ranking by strength. **(C)** Ranking by duration.

A keyword network diagram, constructed with VOSviewer (**Figure 9**), visualizes these findings. The top 22 keywords exhibiting citation bursts were identified using CiteSpace, ranked by duration, start time, and burst intensity (**Figure 10**). Periods spanning 2014 to 2023 are represented by green lines, with burst cycles marked by red lines.

Angiogenesis emerged as the earliest burst keyword in 2014 (**Figure 10A**), reflecting early and sustained interest in its role in immunotherapy and targeted therapy for HCC.

The keyword “Phase III” showed the most intense burst, highlighting a significant focus on this stage of clinical trials (**Figure 10B**).

“Postoperative recurrence” demonstrated the longest period of sustained citation bursts (**Figure 10C**), indicating ongoing research interest in recurrence after surgical intervention.

The 22 burst keywords were classified into two primary categories: body immunity relevant to HCC and immunotherapy for HCC. Between 2018 and 2021, the concentration of burst keywords increased, suggesting that research on targeted immunotherapy for HCC is becoming more specialized, as illustrated by the keyword timeline (**Figure 8**).

## 4 Discussion

Researchers from mainland China lead global publications on immunotherapy and targeted therapy combinations for HCC treatment, contributing 40% of the total output. Several factors underpin this dominance. First, China bears a significant liver cancer burden, with around 55% of global cases attributed to hepatitis B infections (1), compounded by widespread aflatoxin contamination (16). Additionally, regional, economic, and socio-cultural factors, including dietary habits and historical medical practices—such as the reuse of syringes during vaccination campaigns in the 1990s—further intensify this burden. Second, the Chinese government has actively promoted immunotherapy research, establishing vital infrastructure and providing financial support to advance the field, particularly targeting hepatitis B and its liver cancer risks. Japanese researchers rank second in publication volume, facing similar hepatitis B infection challenges (17). Substantial government investment in health research, along with dietary habits such as the predilection of raw food, contributes to Japan’s research output. However, both nations display low centrality, indicating limited academic visibility and influence, highlighting the need for improved publication quality and increased international collaboration.

Among the top 10 publishing countries, France exhibits the highest centrality, reflecting its substantial academic influence and extensive global collaboration in liver cancer research.

American scholars rank third in both publication volume and centrality in the field of immunotherapy and targeted therapy combinations for HCC. While the incidence of liver cancer in the United States remains relatively low compared to countries with higher rates, this is likely due to the country’s advanced healthcare system, comprehensive health education, and robust medical security infrastructure. Nevertheless, the large population base in the U.S. results in a substantial number of patients with liver cancer. As lifestyle changes and the aging population contribute to rising incidence rates, the prevalence of liver cancer in the U.S. is expected to increase. Key factors driving the rise of HCC include obesity, diabetes, and the growing incidence of non-alcoholic fatty liver disease (NAFLD). Additionally, disparities in healthcare access, preventive measures like hepatitis vaccinations, and early screening disproportionately impact vulnerable populations. Racial and ethnic minorities, such as Blacks and Native Americans, face a higher risk of liver cancer compared to Whites, with these disparities potentially linked to genetic, environmental, and socioeconomic factors.

The increasing number of publications on immunotherapy and targeted therapy combinations reflects a growing interest in this approach as a promising treatment for liver cancer. This upward trend not only facilitates the accumulation and dissemination of knowledge but also promotes international collaboration and the exchange of insights, driving further exploration and innovation. The expanding body of research signals a positive outlook for the field, offering more precise treatment options for clinical practice, improving survival rates for patients with HCC, and providing valuable theoretical guidance for clinical management.

Recent advancements in liver cancer treatment have been marked by significant breakthroughs through the combination of molecular targeted therapy and immune checkpoint inhibitors. Clinical trial outcomes have demonstrated sustained clinical benefits, with manageable efficacy and safety profiles (18). Moreover, the development of new drugs is expected to lead to the emergence of more innovative combination regimens in the future (19, 20). In addition to the established combination of targeted therapy and immunotherapy, clinical practice has gradually incorporated other combinations, such as targeted therapy combined with local treatments (21, 22). These regimens, which pair targeted drugs with immunotherapy, offer more precise approaches to liver cancer treatment. This combination strategy can more effectively reduce tumor size and number, thereby increasing patient survival rates and improving quality of life. As new targeted drugs and immunotherapies continue to evolve, treatment outcomes are anticipated to further improve.

With ongoing technological advancements and deeper research, the future of targeted immunotherapy for liver cancer holds promising prospects. Future research can leverage bibliometric analysis to identify existing knowledge gaps and steer subsequent investigations. As understanding of targeted immunotherapy for HCC deepens, the development of more effective, tailored treatment approaches is expected to improve the prognosis and quality of life for individuals diagnosed with liver cancer.

The systemic treatment of HCC has transitioned from single-agent targeted therapies to dual immunotherapies or combined targeted and immunotherapy regimens. Among these, the combination of PD-1/PD-L1 inhibitors with MEK inhibitors has become a major focus of research. Multiple Phase III studies, including the prospective randomized controlled STORM study (23), the international multicenter IMbrave050 study, which met its primary endpoint in a pre-specified interim analysis (24), and ongoing Phase III trials such as JUPITER-04, EMERALD-2, and KEYNOTE-937, suggest that “clinical research” will remain a dominant topic in this field. However, as Phase III studies progress, concerns have emerged regarding the cumulative toxicity of combined targeted and immunotherapy regimens, which has increased the incidence of severe treatment-related adverse events (25, 26). This trend underscores the need for careful monitoring and potential treatment interruptions due to adverse events.

This study has several limitations. Firstly, while the current research status is adequately described, the analysis relies solely on data from the WOS database, introducing potential bias due to the use of a single data source. Secondly, the inclusion criteria limited studies to English-language publications, which may result in language bias by excluding relevant research published in other languages. Additionally, disparities in national wealth and population size may contribute to research bias, as these factors influence a country’s investment in health research. Finally, bibliometric analyses often suffer from temporal biases, as publications with lower citation counts in their early stages may be undervalued despite their high quality. Thus, ongoing attention to emerging studies and publications in diverse languages is crucial for capturing current, valuable insights.

It is anticipated that more research on immunotherapy and targeted therapy combinations for HCC treatment will be published in the coming years, reflecting the rapid development in this field. Future research hotspots are likely to focus on the pathological mechanisms of liver cancer, the development of new drugs, and the design of novel combination regimens. In conclusion, the combination of immunotherapy and targeted therapy represents a promising and emerging treatment modality for liver cancer, with significant potential to improve patient prognosis and quality of life.

## Data Availability

The raw data supporting the conclusions of this article will be made available by the authors, without undue reservation.
