# Supplementary material for: Comprehensive analysis of publications concerning combinations of immunotherapy and targeted therapies for hepatocellular carcinoma: a bibliometric study
Source: Front Immunol. 2025 Feb 12;16:1476146. doi: 10.3389/fimmu.2025.1476146 (PMC11860873; doi:10.3389/fimmu.2025.1476146)
Supplement: Supplementary file 1 [file Table1.docx]

|  | Term | Date | Retrieval results |
| --- | --- | --- | --- |
| #1 | TI=(liver carcinoma) OR TI=(liver cancer) OR TI=(liver tumor) OR TI=(Liver neoplasms) OR TI=(Hepatocellular Carcinoma) OR TI=(hepatocellular cancers) OR TI=(Hepatic tumor) OR TI=(Hepatic cancer) OR TI=(hepatic neoplasms) OR TI=(Hepatoma) OR TI=(Hcc) OR TI=(cancer of the liver) OR TI=(cancer of liver) OR TI=(Neoplasm of liver) OR TI=(liver and intrahepatic bile duct carcinoma) OR TI=(liver and intrahepatic biliary tract cancer) OR TI=(Tumor of liver) OR TI=(liver malignant tumors) OR TI=(Neoplasm of the liver) OR TI=(Hepatocellular Carcinomas) OR TI=(liver cell carcinoma) OR TI=(liver cell carcinomas) OR TI=(hepatic malignancy) OR TI=(liver malignancy) OR TI=(hepatocellular malignancy) OR TI=(malignancy of liver) OR TI=(carcinoma of liver) OR TI=(hepatocellular neoplasm) OR TI=(hepatocellular neoplasia) OR TI=(liver neoplasia) OR TI=(hepatic neoplasia) OR TI=(Hepatoma) OR TI=(Hepatomas) | 1990-01-01  ——  2023-12-31 | 144887 |
| #2 | TI=(Molecular Targeted Therapy) OR TI=(Tyrosine Protein Kinase Inhibitors) OR TI=(Protein Kinase Inhibitors) OR TI=(targeted therap*) OR TI=(targeted molecular*) OR TI=(drugs targeting) OR TI=(tyrosine kinase inhibitor*) OR TI=(Protein Kinase) OR TI=(kinase inhibitor*) OR TI=(targeted therapy) OR TI=(targeted therapies) OR TS=(Sorafenib) OR TI=(Sorafenib) OR TS=(Bevacizumab) OR TI=(Bevacizumab) OR TS=(Donafenib) OR TI=(Donafenib) OR TS=(Lenvatinib) OR TI=(Lenvatinib) OR TS=(Sorafenib) OR TI=(Sorafenib) OR TS=(Bevacizumab) OR TI=(Bevacizumab) OR TS=(Donafenib) OR TI=(Donafenib) OR TS=(Lenvatinib) OR TI=(Lenvatinib) | 1990-01-01  ——  2023-12-31 | 273740 |
| #3 | TI=(Immunotherapy) OR TI=(antibodies, monoclona) OR TI=(immunotherap*) OR TI=(monoclonal antibod*) OR TI=(Atezolizumab) OR TS=(Atezolizumab) OR TI=(Sintilimab) OR TS=(Sintilimab) OR TI=(CTLA-4) OR TS=(CTLA-4) OR TI=(cytotoxic T lymphocyte-associated antigen-4) OR TS=(cytotoxic T lymphocyte-associated antigen-4) OR TI=(PD-1) OR TS=(PD-1) OR TI=(immune checkpoint blockade) OR TS=(immune checkpoint blockade) OR TI=(immune-checkpoint blockade) OR TS=(immune-checkpoint blockade) OR TI=(immune checkpoint inhibitor) OR TS=(immune checkpoint inhibitor) OR TI=(immune-checkpoint inhibitor) OR TS=(immune-checkpoint inhibitor) OR TI=(ICI) OR TS=(ICI) OR TI=(ICIs) OR TS=(ICIs) OR TI=(CPI) OR TS=(CPI) OR TI=(PD-L1) OR TS=(PD-L1) | 1990-01-01  ——  2023-12-31 | **217485** |
| #4 | #1 AND #2 AND #3 |  | **2179** |
| #5 | #1 AND #2 AND #3 and Science Citation Index Expanded (SCI-EXPANDED) (Web of Science index) and  English (Languages)  And Artical or Review Artical or Meeting Abstract or Proceeding Paper (Document Types) |  | **1933** |
